# Rapid optical determination of β-lactamase and antibiotic activity

**DOI:** 10.1186/1471-2180-14-84

**Published:** 2014-04-04

**Authors:** Shazia Khan, Ulysses W Sallum, Xiang Zheng, Gerard J Nau, Tayyaba Hasan

**Affiliations:** 1 Wellman Centre for Photomedicine, Massachusetts General Hospital, Harvard Medical School, Boston, MA 02114, USA; 2 Department of Microbiology and Molecular Genetics, Department of Medicine, Division of Infectious Diseases, Centre for Vaccine Research, University of Pittsburgh School of Medicine, Pittsburgh, PA 15261, USA; 3 Present address: Partners Research Ventures and Licensing, 101 Huntington Ave., 4th Floor, Boston, MA 02199, USA; 4 Present address: Pfizer Inc, 401 N Middletown Rd, Pearl River, NY 10965, USA

**Keywords:** Fluorescence, Fluorophore, Quenching, Staphylococcus, Cephalosporin, Cefazolin, β-lactamase, β-lactam, Antibiotic activity, Antibiotic susceptibility

## Abstract

**Background:**

The absence of rapid tests evaluating antibiotic susceptibility results in the empirical prescription of antibiotics. This can lead to treatment failures due to escalating antibiotic resistance, and also furthers the emergence of drug-resistant bacteria. This study reports a rapid optical method to detect β-lactamase and thereby assess activity of β-lactam antibiotics, which could provide an approach for targeted prescription of antibiotics. The methodology is centred on a fluorescence quenching based probe (β-LEAF – **β-L**actamase **E**nzyme **A**ctivated **F**luorophore) that mimics the structure of β-lactam antibiotics.

**Results:**

The β-LEAF assay was performed for rapid determination of β-lactamase production and activity of β-lactam antibiotic (cefazolin) on a panel of *Staphylococcus aureus* ATCC strains and clinical isolates. Four of the clinical isolates were determined to be lactamase producers, with the capacity to inactivate cefazolin, out of the twenty-five isolates tested. These results were compared against gold standard methods, nitrocefin disk test for β-lactamase detection and disk diffusion for antibiotic susceptibility, showing results to be largely consistent. Furthermore, in the sub-set of β-lactamase producers, it was demonstrated and validated that multiple antibiotics (cefazolin, cefoxitin, cefepime) could be assessed simultaneously to predict the antibiotic that would be most active for a given bacterial isolate.

**Conclusions:**

The study establishes the rapid β-LEAF assay for β-lactamase detection and prediction of antibiotic activity using *S. aureus* clinical isolates. Although the focus in the current study is β-lactamase-based resistance, the overall approach represents a broad diagnostic platform. In the long-term, these studies form the basis for the development of assays utilizing a broader variety of targets, pathogens and drugs.

## Background

Bacterial drug resistance is a growing global health challenge. Resistant infections are difficult to treat, tend to spread relatively rapidly and increase healthcare costs significantly [1]. Empiric antibiotic therapy is commonly started before the results of antimicrobial susceptibility testing (AST) are available. This is mainly because the available AST methods are slow, typically requiring 24–72 hours, being primarily based on bacterial growth. Inappropriate empiric antibiotic regimens can be associated with treatment failures/prolonged illness [2,3], and may also serve to promote resistant bacterial strains [4-7]. Pre-prescription AST, such as rapid point-of-care diagnostics, that can help identify the most effective antibiotic for bacterial infections would be advantageous, especially in the context of escalating resistance [8-10].

Bacterial antibiotic resistance can be due to a variety of mechanisms, including enzymatic inactivation of antibiotics, altered target sites, decreased uptake and/or increased efflux of the antimicrobial agents [11]. Multiple resistance factors can be present simultaneously [12,13]. β-lactamases are a major antibiotic resistance mechanism against the widely used β-lactam antibiotics, which target penicillin-binding proteins (PBPs) involved in bacterial cell wall synthesis [14]. β-lactamase enzymes inactivate β-lactam antibiotics, by hydrolyzing their β-lactam ring essential to antibiotic function [15,16]. There is a wide array of β-lactamases with varying specificities and activities, and this resistance mechanism has clinical significance [16-18]. Notably, many of the ‘ESKAPE’ pathogens (*Enterococcus faecium,Staphylococcus aureus,Klebsiella pneumonia,Acinetobacter baumanni,Pseudomonas aeruginosa* and *Enterobacter species*), responsible for a majority of nosocomial infections [19], may produce β-lactamases.

Alongside the ever-growing threat of Methicillin Resistant *S. aureus* (MRSA), Methicillin Susceptible *S. aureus* (MSSA) strains are also highly prevalent and responsible for severe infections such as infective endocarditis [20,21]. Both MRSA and MSSA can produce β-lactamases [22-25]. Though by historical definition, expression of an altered target penicillin binding protein PBP2’ with lowered affinity for β-lactam antibiotics results in methicillin resistance [26-28], β-lactamase alone may be responsible for borderline methicillin/oxacillin resistance phenotype even in strains without PBP2’ [29]. Most MRSA strains produce β-lactamase in addition to PBP2’ [22-24]. Among MSSA, ~90% strains are β-lactamase producers [30].

β-lactamases can therefore present a challenge to successful anti-bacterial therapy, in particular where the bacterial burden is high. Cephalosporins are the treatment of choice for MSSA infections [31-33]. Although traditionally cephalosporins were believed to be stable to the *S. aureus* β-lactamases, an ‘inoculum effect’ has been demonstrated, wherein at high inocula some cephalosporins get hydrolysed by β-lactamases [34,35]. The inoculum effect with different cephalosporins has been reported in clinical isolates of MSSA [33,36], and instances of clinical failure of cephalosporins are well documented in high-inoculum staphylococcal endocarditis infections and bacteremia [37-40]. The inoculum effect is not limited to *Staphylococcus*, and is observed in other bacteria including *Enterobacteriaceae, Pseudomonas* and *Neisseria gonorrhoeae*, with antibiotic classes other than cephalosporins as well [35].

Evaluation of antibiotic susceptibility and detection of resistance are mainly performed by means of disk diffusion assays or broth/agar dilution to determine minimum inhibitory concentration (MIC = lowest concentration of antibiotic that inhibits the bacterial growth), where bacteria are cultured in the presence of antimicrobials and respective growth patterns observed [41,42]. Besides agar or broth dilution, the E-test is a relatively new, yet established method for MIC determination, and consists of a predefined gradient of antibiotic concentrations on a plastic strip (http://www.biomerieux-diagnostics.com). The strips are placed on inoculated agar plates and read following incubation, in a manner similar to the disk-diffusion procedure ([43], http://www.biomerieux-diagnostics.com). For all of these tests, based on the results obtained, the bacteria are classified as susceptible, intermediate or resistant to the tested antimicrobial agent using breakpoints, i.e. threshold values put forth by the Clinical and Laboratory Standards Institute (CLSI) or other regulatory authorities [41,42]. These methods rely on growth of bacteria, hence are time-consuming and unable to provide information to guide antibiotic administration until about 24 h after a pathogen has been isolated. They may also prove to be imprecise in antibiotic susceptibility prediction in case of resistant bacteria, especially in context of β-lactamase producers [44,45]. This is because even if the presence of a resistance factor results in altered MICs or disk diffusion diameters, interpretation can remain unaffected, as breakpoints may not be reached [46,47]. To address this issue, the CLSI regularly puts forth revised breakpoints and updates and often recommends additional testing, such as determination of specific resistance mechanisms (e.g. β-lactamase production) [41,42]. Also at times repeated testing may be needed, such as in cases of serious infections requiring penicillin therapy, the CLSI guidelines recommend repeated MIC and β-lactamase testing on all subsequent isolates from patients [41,48]. Given these challenges, new methodologies that can provide timely bacterial resistance and/or antibiotic susceptibility information, such as that developed in our study, would be valuable.

**Figure 1:**
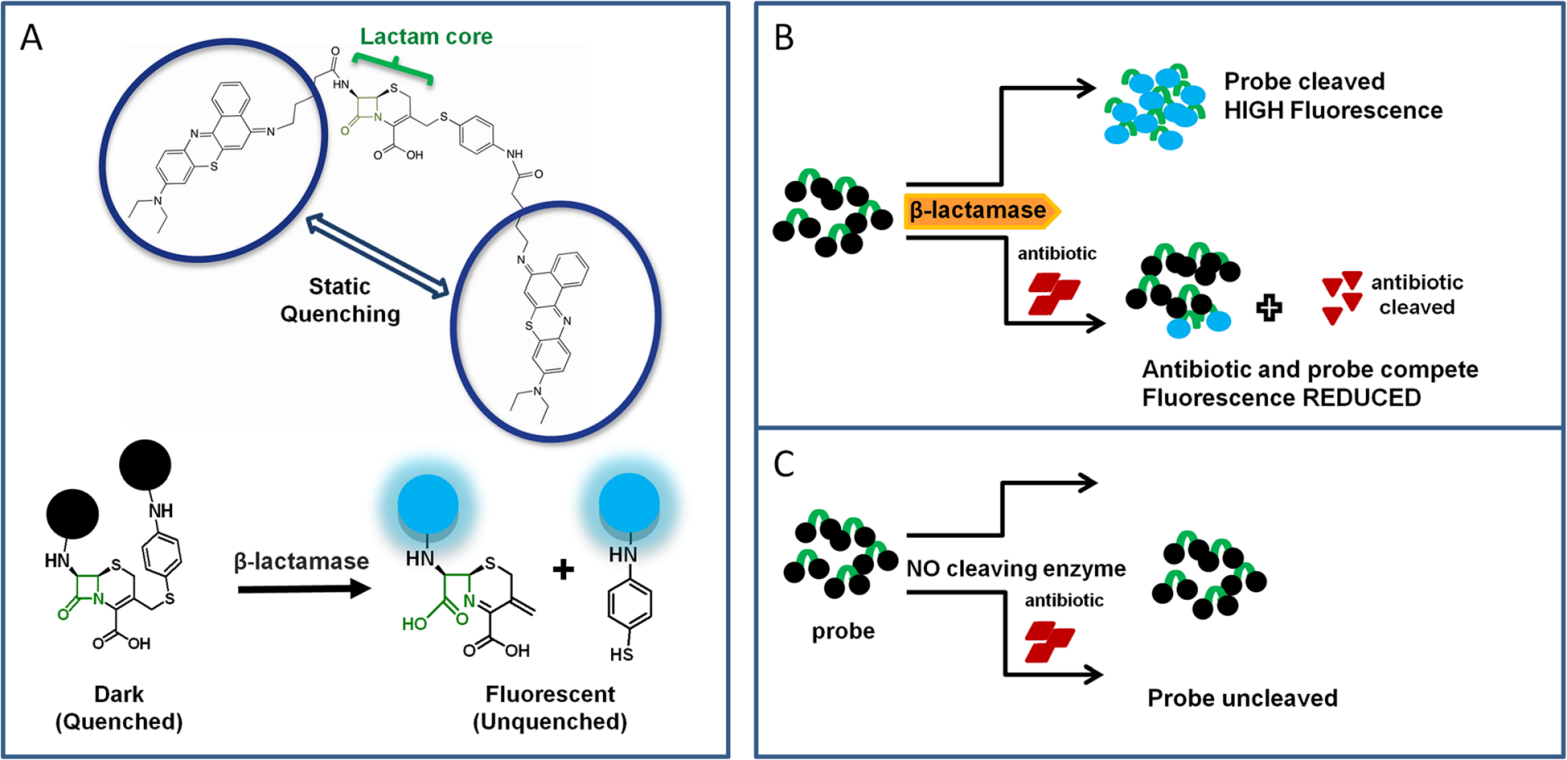
**Schematic showing the principle of the β-LEAF assay. A**. The β-LEAF probe comprises a β-lactam core structure including the cleavable lactam ring (green), flanked by two fluorophores (encircled), which undergo static quenching when the probe is intact. Following cleavage by β-lactamase, the fluorophores move apart and show fluorescence. **B**. Assay profile for β-lactamase producing bacteria **C**. Assay profile for lactamase non-producing bacteria.

In this study we describe a rapid optical method (~60 min) for β-lactamase detection and assessing activity of β-lactam antibiotics in presence of respective β-lactamase (β-lactamase based antibiotic activity). The antibiotic activity may also be interpreted more broadly as antibiotic susceptibility (β-lactamase based antibiotic susceptibility). We have developed a fluorescent molecular probe β-LEAF [β-Lactamase Enzyme Activated Fluorophore (described as β-LEAP in earlier publications)], based on fluorophore quenching-dequenching, for rapid detection and characterization of β-lactamases [49,50]. Although β-lactamase is widely employed as a reporter system for gene expression using fluorescent probes ([51-54] and (http://http:/www.invitrogen.com)), this approach is novel in that it also incorporates assessment to predict the most active β-lactam antibiotic among tested antibiotics, against given bacteria. In a previous report we demonstrated the principle using ATCC strains with known β-lactamase production for rapid functional definition of Extended Spectrum β-Lactamases [50]. In the current study we tested the approach with a panel of MSSA clinical isolates, to determine β-lactamase production and predict the activity of tested β-lactam antibiotic(s), in a rapid assay. The concept behind the β-LEAF assay is illustrated in Figure 1. The prototype β-LEAF construct mimics the structure of β-lactam antibiotics. It contains a cephalosporin (β-lactam) core structure, including a cleavable lactam ring, conjugated to two identical fluorophore (EtNBS) moieties [49]. The two fluorophores flanking the cephalosporin core are in close apposition in the intact probe, which results in static (ground-state) quenching. β-lactamase activity is detected by an increase in fluorescence over time as the enzyme cleaves β-LEAF to generate dequenched fluorophores (Figure 1). When present together, an excess β-lactam antibiotic and β-LEAF compete for the β-lactamase enzyme due to structural similarity, leading to reduced β-LEAF cleavage rate and thus reduced fluorescence change rate, compared to when β-LEAF is present alone (Figure 1B). The reduction in fluorescence provides insight into activity of the tested β-lactam antibiotic in the presence of β-lactamase (β-lactamase-based antibiotic activity). The read-out for the assay is optical (fluorescence), rather than bacterial viability or based on growth of bacteria. We performed the assays with *S. aureus* clinical isolates and cephalosporin antibiotics and validated the results against standard methodologies for β-lactamase and antibiotic susceptibility determination using nitrocefin disk tests and disk diffusion or E-tests respectively. Furthermore, we showed simultaneous testing of multiple antibiotics, to help predict the most suitable antibiotic that could be used for therapy. Though validation in a large number of isolates is needed to establish the robustness of the assay, the initial results in a sample set are encouraging, especially because the method is ~20 times faster than conventional methods. The β-LEAF assay demonstrates the use of fluorescent substrates to rapidly characterize resistance and predict antibiotic activity, and represents the first step towards the development of a broader diagnostic platform.

## Methods

### Reagents, bacterial strains and culture conditions

Brain Heart Infusion (BHI) broth and BHI agar were obtained from BD Difco (BD: Becton, Dickinson and Company, New Jersey, USA). Penicillin disks (10U), cefazolin disks (30 μg), Mueller-Hinton II agar plates for susceptibility testing by agar disk diffusion and cefinase disks (nitrocefin disks) for detection of β-lactamase were purchased from BD BBL. Cefoxitin and cefazolin E-test strips were purchased from bioMerieux (Marcy l’Etoile, France). Cefazolin-sodium and cefoxitin-sodium (powder form) and cefepime-HCl (powder form) were obtained from Novaplus (Novation, Texas, USA) and Sagent Pharmaceuticals (Schaumburg, Illinois, USA) respectively. *S. aureus* strains used in this study were purchased from ATCC (Manassas, Virginia, USA) and clinical isolates were provided by Dr. M.J. Ferraro (Microbiology Labs, Massachusetts General Hospital, Boston, MA, USA) (Table 1). All strains were routinely cultured in BHI agar or broth at 37°C. The isolates were grown in presence of penicillin disks to induce and enhance β-lactamase production as required. For the disk diffusion assays, Mueller-Hinton II agar plates were incubated at 35°C.

**Table 1:** ***S. aureus *** **isolates used in the study and their β-lactamase genotype and phenotype**

**#**	***S. aureus *** **isolate**	β-lactamase genotype*^&^**(‘*blaZ’ *** **PCR)**	**β-lactamase phenotype by nitrocefin disk test**
1	29213	Positive	+
2	25923	Negative	**-**
3	75391-09	Positive	-
4	W5337	Negative	-
5	W53156	Positive	-
6	AI5070237	Positive	+
7	AI5081845	Positive	-
8	159570-08	Positive	-
9	H30876	Positive	-
10	32455-09	Positive^$^	-
11	HIP12052	Positive	-
12	AI5090298	Positive	-
13	F33263-2	Positive	-
14	AI5090297	Positive	-
15	HIP11033	Positive	-
16	HIP11353	Positive^$^	-
17	158390-08	Positive^$^	-
18	F52670	Positive	+
19	H63189	Positive	+
20	M24125	Positive	+
21	F20358.1	Negative	-
22	H67147.3	Positive	-
23	M60028	Negative	-
24	KI58249.2	Unknown	-
25	M69678	Negative	-
26	X33116	Positive	-
27	F29916-2	Positive	-

*S. aureus* strains 29213 (#1) and 25923 (#2) were obtained from ATCC and the *S. aureus* clinical isolates (#3 - #27) were provided by Dr. Mary Jane Ferraro (Microbiology Labs, Massachusetts General Hospital, Boston, MA, USA). Isolate numbers (e.g. #1 for 29213, etc) are used to refer to the different isolates throughout the study.

*The β-lactamase genotype was determined by PCR to detect *blaZ* (staphylococcal β-lactamase gene). Genotype data for isolates #3 - #15 was kindly provided by Dr. Robert L. Skov, Statens Serum Institut (R. L. Skov, unpublished results) and for #16 - #27 by Dr. Mary Jane Ferraro.

^&^All isolates are MSSA.

^$^Special comment – *blaZ* contained Stop codon or deletion (so non-functional) (R. L. Skov, unpublished results).

Nitrocefin disk test to determine β-lactamase production was performed as described in Methods. Development of orange colour uniformly, similar to positive control #1, was taken as positive reaction, indicated by ‘+’ symbols. ‘-’ denotes negative result (i.e. no colour change). The results are representative of three independent experiments, which gave consistent results.

### β-LEAF synthesis

β-LEAF was synthesized as previously described [49]. Briefly, the chloro- group on 7-amino-3-chloromethyl-3-cephem-4-carboxylic acid *p*-methoxybenzyl ester (ACLE) was substituted with 4-aminothiophenol with the help of 4-methylmorpholine. The purified product was mixed with 5-(4′-carboxybutylamino)-9-diethylaminobenzo[a]phenothiazinium chloride (EtNBS-COOH), O–(7-azabenzotriazole-1-yl)-*N,N,N,N*’-tetramethyluroniumhexafluorophosphate(HATU), and diisopropylethylamine in dry *N,N*-dimethylformamide. The reaction mixture was stirred at room temperature for 3 h then purified on silica coated preparative thin-layer chromatography. After removal of the *p*-methoxybenzyl protection group, Reversed Phase-High Performance Liquid Chromatography was performed to yield β-LEAF in high purity (>95%). Concentrated stocks were prepared in 100% DMSO and stored at −20°C.

### β-LEAF- antibiotic fluorescence assay

Bacterial strains were cultured on BHI agar plates in the presence of a penicillin disk (10U) overnight. For each bacterial isolate, colonies closest to the penicillin disk were transferred to PBS to make a homogenous suspension [~10^9^ Colony Forming Units (CFU)/ml]. Bacterial O.D. was measured at 600 nm. 100 mM antibiotic solution (4X stock) was prepared by dissolving the antibiotic powder in PBS, and 20 μM β-LEAF probe solution (2X stock) was prepared in 40% DMSO in PBS. The assays were performed in 96-well white clear-bottom plates in a total volume of 100 μl respectively, to include bacteria and 10 μM β-LEAF probe, with or without 25 mM antibiotic (cefazolin). Each reaction was set up as follows: 25 μl bacterial suspension, 25 μl antibiotic 4X stock solution or PBS only and 50 μl probe 2X stock solution, with resultant buffer concentration as 20% DMSO in PBS in each 100 μl reaction. For each isolate, reactions were performed in triplicate in the absence and presence of test antibiotic respectively. Time course assays were carried out, monitoring β-LEAF cleavage by measuring fluorescence for 60 min, at 1 min intervals (Spectramax M5 Plate Reader, Molecular Devices). Instrument settings were kept as excitation 640 nm, emission 700 nm and temperature was maintained at 37°C throughout. β-LEAF cleavage rate in each case was determined as slope i.e. fluorescence change as a function of time (obtained from instrument software - SoftMax Pro5), normalized by bacterial O.D.

For multiple antibiotic testing, reactions were similarly set up with β-LEAF only, and with β-LEAF and cefazolin, cefoxitin or cefepime in separate reactions.

*S. aureus* ATCC strains with established β-lactamase status, β-lactamase producing strain 29213 (#1), and β-lactamase negative strain 25923 (#2), were used as positive and negative control strains respectively in all assay sets. Bacteria-free controls (PBS only) were also included in each assay set.

For ‘un-induced’ growth cultures, bacterial strains/isolates were cultured on non-selective BHI agar plates, with the rest of the protocol remaining unchanged.

### Nitrocefin disk test for detection of β-lactamase

The experiments were performed using cefinase disks (nitrocefin disks) as per manufacturer’s recommendations. Briefly, *S. aureus* isolates grown on agar plates in the presence of penicillin disks (to induce and enhance β-lactamase production) respectively were used. For each isolate, the nitrocefin disks were moistened with ddH_2_O, and colonies that grew closest to the penicillin disk were smeared evenly across the nitrocefin disk surface using an inoculating loop. Disks were observed for colour change up to 60 min. β-lactamase producer strain ATCC 29213 (#1) and β-lactamase negative strain ATCC 25923 (#2), were used as positive and negative controls respectively.

### Antibiotic susceptibility testing - disk diffusion and E-test

The standard procedure recommended by CLSI was followed [41,42]. Briefly, inoculum was prepared by the direct colony suspension method preferred for *S. aureus.* Isolated colonies from non-selective overnight BHI agar plates were used to make a saline suspension, and turbidity was adjusted equivalent to a 0.5 McFarland turbidity standard. Thereafter, the standardized inoculum was spread uniformly on a Mueller Hinton II agar plate, allowed to dry, cefazolin disk applied to the centre of the plate, and plates incubated at 35°C for 20–24 h. The zones of inhibition were measured and compared against CLSI Zone Diameter Interpretive Charts, to categorize isolates as susceptible, intermediate or resistant. (The CLSI 2012 charts were used, which were most current at the time of the experiments [41]). *S. aureus* ATCC 25923 (#2) was included in each experiment as the CLSI recommended quality control strain for disk diffusion [41].

For the zone edge test comparison criteria, ATCC 29213 (#1) and ATCC 25923 (#2) were used as the CLSI recommended positive and negative controls, showing ‘sharp’ and ‘fuzzy’ inhibition zone edges respectively.

For the E-test, cefoxitin or cefepime E-test strip was applied to the inoculated plate, and following incubation at 35°C for 24 h, the MIC value was read. The CLSI interpretive criteria, most current at the time of experiments, were used to categorize isolates as susceptible, intermediate or resistant [41]. *S. aureus* ATCC 29213 (#1) was included in each experiment as the recommended quality control for MIC determination [41].

Experiments were similarly performed with ‘induced’ growth cultures, wherein bacteria grown in presence of penicillin disks overnight were used as the starting inoculum to prepare the saline suspension. The standard procedure described above was followed.

## Results

### β-LEAF assays determine β-lactamase production and assess cefazolin activity

We used a panel of *S. aureus* comprising two ATCC strains and 25 clinical isolates (Table 1) as a model system. Isolate numbers (eg. #1, #4, etc.), rather than full names, are used to refer to isolates as per Table 1 throughout this study. ATCC strains with established β-lactamase status, β-lactamase producing strain 29213 (#1) and β-lactamase negative strain 25923 (#2) were used as positive and negative controls respectively. Cefazolin, a first generation cephalosporin, was used as the test antibiotic in these experiments. Each isolate was assayed under two conditions, with β-LEAF alone and with β-LEAF and saturating concentration of cefazolin (2500-fold higher concentration of cefazolin than β-LEAF) respectively.

**Figure 2:**
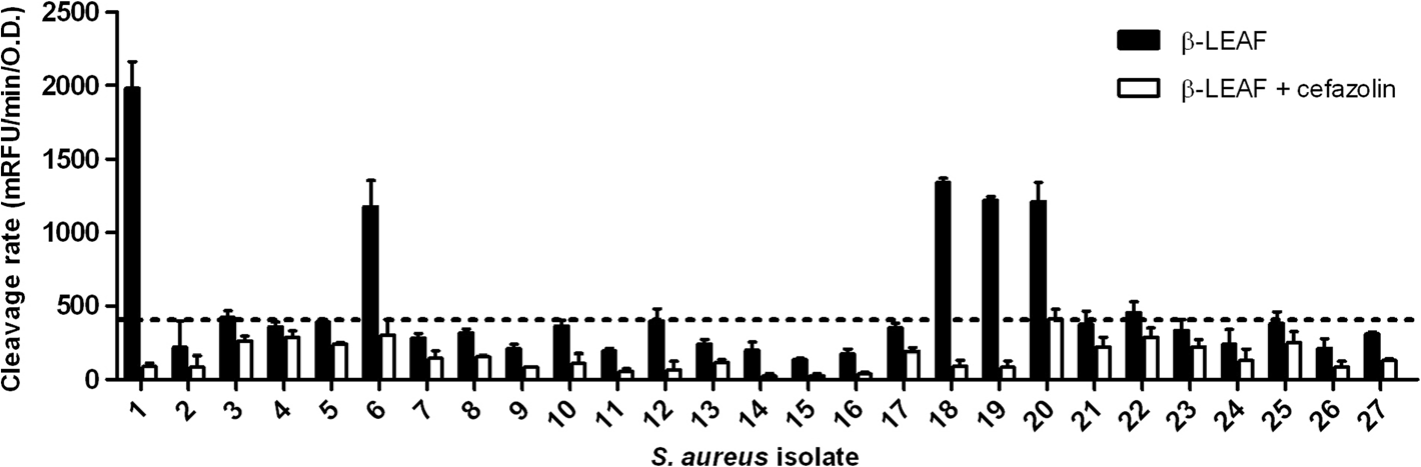
**β-LEAF assays determine β-lactamase production and cefazolin activity in *S. aureus *clinical isolates.** β-LEAF assays were performed with two ATCC *S. aureus* control strains (known β-lactamase producer #1 and non-producer #2) and 25 *S. aureus* clinical isolates, with cefazolin as a test antibiotic. The different bacterial isolates were incubated with β-LEAF (probe) alone and β-LEAF and cefazolin respectively, and fluorescence was monitored over 60 min. The y-axis represents the cleavage rate of β-LEAF (measured as fluorescence change rate – milliRFU/min) normalized by bacterial O.D. (optical density) at 600 nm. The black bars depict cleavage rate when β-LEAF alone is used, to show β-lactamase production. The white bars depict cleavage rate of probe when both the probe and cefazolin are included in the reactions. The horizontal line indicates a proposed cut-off value (upper limit of mean ± 3X Std. deviation for strain #2, β-LEAF probe reaction) to demarcate β-lactamase production. Where the black and white bars are significantly different, the antibiotic is predicted to be less active. Results are presented as the average of three independent experiments (each experiment contained samples in triplicates) and error bars represent the standard error for all isolates, except #2. For #2, the error bar is 3X standard deviation.

Distinct fluorescence profiles were observed for the control strains #1 and #2 (Figure 2). When assayed only in the presence of β-LEAF, a significant increase in fluorescence was observed with the β-lactamase producer strain #1. However, when the assay included both β-LEAF and cefazolin, a drastically lower β-LEAF cleavage rate (as measured by fluorescence change over time) was seen (Figure 2). Strain #2 does not encode β-lactamase and showed low fluorescence in both the β-LEAF alone and β-LEAF + cefazolin reactions (Figure 2).

The various clinical isolates showed different patterns of fluorescence, and were categorized by comparing with the profile of the control strains. When assayed with β-LEAF alone, isolates #6, #18, #19 and #20 showed appreciable β-LEAF cleavage rates similar to that observed for #1 (Figure 2), and were designated as β-lactamase producing strains. These also showed significantly lower cleavage rates when the assay was performed with both β-LEAF and cefazolin (Figure 2). Testing with several-fold higher concentration of the antibiotic compared to probe concentration (as per assay design) increases chances of the antibiotic becoming the preferred substrate for the respective lactamase enzyme. The corresponding decrease in β-LEAF cleavage in the presence of the antibiotic, compared to when β-LEAF is present alone i.e., reduction in fluorescence due to competition (Figure 1), is used to predict activity of the antibiotic (reduction in fluorescence is inversely proportional to its predicted activity in presence of a lactamase). For isolates #6, #18, #19 and #20, the drastically reduced probe cleavage rates (in presence of the antibiotic) indicated that cefazolin was capable of competing against the β-LEAF for enzyme binding, and likely to be a substrate of the β-lactamase in these isolates. Hence, cefazolin may be readily inactivated by the respective lactamases produced by these isolates. All other isolates showed fluorescence profiles similar to #2. Although, ideally #2 should not exhibit fluorescence change over time, a slight increase was noted (Figure 2). A range of mean ±3X standard deviation observed for #2 (β-LEAF only reaction) would give 99.7% confidence intervals for values by Gaussian statistics. The upper limit of this range, i.e. mean + 3X standard deviation was set up as a cut-off value (Figure 2). Isolates showing cleavage rates within this cut-off, that is, low/negligible increase in fluorescence of β-LEAF with time similar to non-producer #2, were designated as non-producers of β-lactamase. Also as negligible differences between the cleavage rates of β-LEAF and β-LEAF + cefazolin reactions were observed, cefazolin was predicted to be active to treat infections caused by these bacteria. Isolates that showed cleavage rate of β-LEAF alone higher than the cut-off included those observed to cleave β-LEAF efficiently (#6, #18, #19 and #20), as well as some isolates showing marginal differences from #2, such as #22. These could be low producers. As the difference in cleavage rates in the absence and presence of cefazolin was minimal in these marginal cases, cefazolin was predicted as active. The results of the β-LEAF assay for all isolates are summarized in Table 2 (column 2 and column 6).

**Table 2:** Comparison of different methods of β-lactamase detection and cefazolin antibiotic susceptibility/activity determination

***S. aureus *** **isolate #**	**β-LACTAMSE GENOTYPE (‘*blaZ’ *** **PCR)**	**β-LACTAMASE PHENOTYPE**			**CEFAZOLIN SUSCEPTIBILITY/ACTIVITY**	
		β-LEAF assay*	**Nitrocefin disk test**	**Zone edge test**	**Disk diffusion**	Antibiotic activity – β-LEAF assay**
	**‘+’ = positive PCR**		**Uniform orange color = ‘+’ (positive)**	**Sharp zone edge = ‘+’ (positive)**	**S = susceptible**	**LA = less active**
	**$: contained stop codon or deletion**				**(!) = sharp zone edge**	**A = active**
1	+	+	+	+	S (!)	LA
2	-	**-**	**-**	**-**	S	A
3	+	-	-	-	S	A
4	-	-	-	-	S	A
5	+	-	-	-	S	A
6	+	+	+	+	S (!)	LA
7	+	-	-	-	S	A
8	+	-	-	-	S	A
9	+	-	-	-	S	A
10	+^$^	-	-	-	S	A
11	+	-	-	-	S	A
12	+	-	-	-	S	A
13	+	-	-	-	S	A
14	+	-	-	-	S	A
15	+	-	-	-	S	A
16	+^$^	-	-	-	S	A
17	+^$^	-	-	-	S	A
18	+	+	+	+	S (!)	LA
19	+	+	+	+	S (!)	LA
20	+	+	+	+	S (!)	LA
21	-	-	-	-	S	A
22	+	(Weak) +	-	-	S	A
23	-	-	-	-	S	A
24	Unknown	-	-	-	S	A
25	-	-	-	-	S	A
26	+	-	-	-	S	A
27	+	-	-	-	S	A
	**Col. 1**	**Col. 2**	**Col. 3**	**Col. 4**	**Col. 5**	**Col. 6**

^$^Special comment – *blaZ* contained Stop codon or deletion (so non-functional) (Robert L. Skov, unpublished results).

*Classification into positive and negative is based on proposed cut-off depicted in Figure 2 (upper limit of mean ± 3X Std. deviation for strain #2, β-LEAF probe reaction) to demarcate β-lactamase production. Isolates showing cleavage rates of β-LEAF (black bars in Figure 2) lower than or equal to the cut-off were designated ‘negative’, while isolates with higher cleavage rates were designated ‘positive’.

**Classification of cefazolin as ‘active’ or ‘less active’: When difference in cleavage rates (fluorescence change) in the absence and presence of cefazolin was minimal, antibiotic predicted to be ‘active’. Drastically lowered cleavage rate in presence of cefazolin compared to when probe assayed alone led to prediction of cefazolin as ‘less active’ respectively (also see Figure 2).

Details of Disk Diffusion results are presented in Table 3.

Bacteria-free controls (PBS only) were included in each assay-set to account for non-specific probe cleavage that may occur. As expected, a negligible fluorescence change over time was observed. Comparison of cleavage rates (mRFU/min) for #1, #2 and the PBS only control are shown in Additional file 1: Figure S1.

### Nitrocefin test for detection of β-lactamase validates results from β-LEAF assay

In order to validate the β-lactamase phenotypes determined by the β-LEAF assay, a CLSI recommended β-lactamase screening method, the chromogenic nitrocefin test, was utilized [41]. All bacterial isolates that were strongly positive by the β-LEAF assay were also found to be positive by nitrocefin conversion with the nitrocefin disks, showing a change in colour from yellow to deep orange in a positive reaction for β-lactamase (Table 1, right-most column).

### Comparison of conventional disk diffusion and β-LEAF assay results

In order to compare predictions of cefazolin activity by the β-LEAF assay to a conventional AST method, we performed cefazolin disk diffusion assays with the *S. aureus* isolates. Based on respective zone of inhibition diameters, each isolate was classified as susceptible, intermediate or resistant using the CLSI zone interpretive criteria (Table 3, Additional file 2: Figure S2). Interestingly, all the isolates fell in the cefazolin ‘susceptible’ range with this methodology (Table 3).

**Table 3:** Cefazolin disk diffusion results

***S. aureus *** **isolate #**	**Zone of inhibition diameter (mm)**	AS*	**Zone edge**	**Interpretation as per zone edge test criteria**^**&**^
1	21.5 ± 1.0	S	Sharp	β
2	31.0 ± 1.0	S	Fuzzy	
3	33.5 ± 0.5	S	Fuzzy	
4	33.0 ± 2.0	S	Fuzzy	
5	32.5 ± 0.5	S	Fuzzy	
6	36.5 ± 0.5	S	Sharp	β
7	32.0 ± 0.5	S	Fuzzy	
8	39.5 ± 1.5	S	Fuzzy	
9	29.5 ± 1.5	S	Fuzzy	
10	41.5 ± 0.5	S	Fuzzy	
11	34.5 ± 2.5	S	Little fuzzy	Weak β?
12	41.0 ± 1.6	S	Fuzzy	
13	32.5 ± 0.5	S	Fuzzy	
14	33.0 ± 0.0	S	Fuzzy	
15	35.5 ± 2.5	S	Fuzzy	
16	36.5 ± 0.5	S	Fuzzy	
17	36.5 ± 0.5	S	Fuzzy	
18	33.5 ± 0.5	S	Sharp	β
19	31.0 ± 0.0	S	Sharp	β
20	20.5 ± 0.3	S	Sharp	β
21	38.0 ± 1.0	S	Fuzzy	
22	34.0 ± 1.1	S	Little fuzzy	Weak β?
23	33.5 ± 1.5	S	Fuzzy	
24	34.5 ± 1.5	S	Fuzzy	
25	30.5 ± 0.5	S	Fuzzy	
26	34.0 ± 0.0	S	Fuzzy	
27	36.0 ± 2.0	S	Little fuzzy/sharpish	Weak β?

*The Antibiotic Susceptibility (AS) was determined using the CLSI Zone Diameter Interpretive Criteria for Cefazolin Disk Diffusion [41].

≤ 14 mm: Resistant (R); 15–17 mm: Intermediate (I); ≥ 18 mm: Susceptible (S)

The results shown are averages of at least two independent experiments, and are presented as Average ± Standard Error. The CLSI recommended quality control strain ATCC 25923 (#2) was included each time and gave zone of inhibition diameter within the expected range (29-35 mm) [41].

^&^The zone edge test was also applied and the edge of the zone of inhibition was observed. *S. aureus* ATCC 29213 (#1) was used as a positive control for the zone edge test (sharp edge), and ATCC 25923 (#2) as a negative control (fuzzy edge). ‘β’ denotes β-lactamase producing strain.

To ascertain whether isolates producing detectable amounts of β-lactamases would show altered disk diffusion results, we performed disk-diffusion assays for the predicted ‘cefazolin less active’ isolates (#1, #6, #18, #19, #20) (Figure 2) of the β-LEAF assay using both ‘induced’ and ‘un-induced’ growth cultures as inoculum respectively (conventional AST is usually performed using ‘un-induced’ inoculums). This would also verify if observed discrepancy in antibiotic activity/susceptibility prediction between the β-LEAF assay and disk-diffusion was caused by the different induction statuses (β-LEAF assay = induced growth cultures, disk diffusion assays = standard growth, see Methods). Using induced cultures as starting inoculum, however, did not change the results of cefazolin AST, compared to using standard (un-induced) inoculum (Additional file 3: Table S1).

β-lactamase detection is an important screening test, and the zone edge test (using penicillin) has recently been included in the CLSI guidelines for this purpose. [41,42]. A sharply demarcated zone edge in disk diffusion assays correlates well with β-lactamase production [41,42,55]. Based on this criterion, a sharp zone edge for isolates #1, #6, #18, #19, and #20 was seen, designating them lactamase producers (Table 3, Additional file 2: Figure S2). The same set of isolates was predicted to be ‘cefazolin less active’ and lactamase producers using the β-LEAF assay and nitrocefin tests (Figure 2, Table 1 (nitrocefin test results), Table 2). Thus, the disk-diffusion test results on the whole, with results from cefazolin susceptibility and zone edge tests taken together, corresponded with the β-LEAF assay predictions, as by virtue of β-lactamase production respective isolates may show some degree of resistance to cefazolin.

Table 2 summarises comparison of results for β-lactamase production (columns 2–4) and cefazolin susceptibility/activity (columns 5–6), along with the β-lactamase genotypes (column 1) for all isolates in the study. Overall, the results from the rapid β-LEAF assay were consistent with results from the standard methods, validating the methodology. However, the presence of the *blaZ* gene did not always correlate with a lactamase positive phenotype.

### Defining activity profiles for multiple antibiotics using β-LEAF

**Figure 3:**
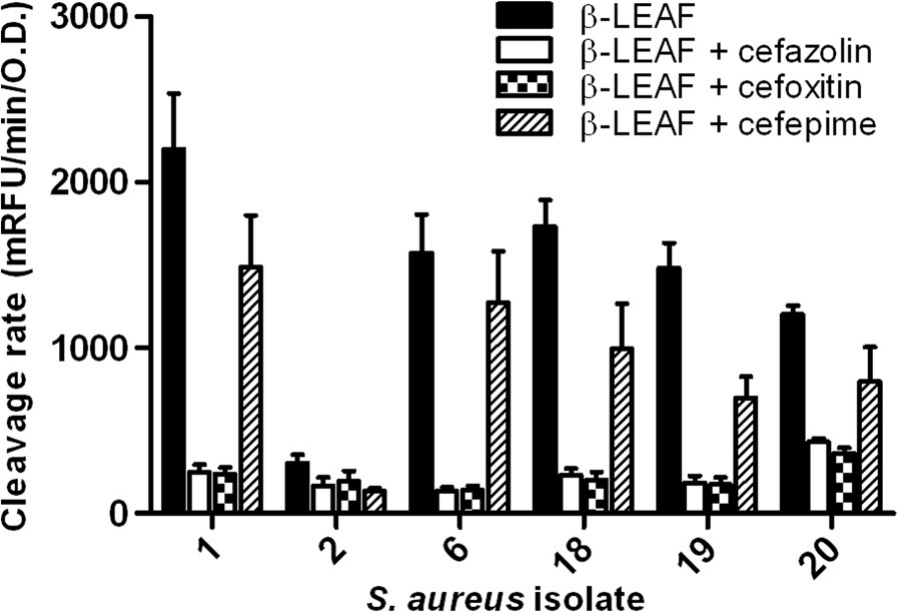
**β-LEAF assays can be used to determine activity of multiple antibiotics simultaneously.** β-LEAF assays were set up with multiple antibiotics (cefazolin, cefoxitin and cefepime) in selected *S. aureus* isolates. Antibiotic activity was assessed in positive control strain #1, negative control strain #2 and four *S. aureus* clinical isolates that showed substantial β-lactamase production (#6, #18, #19, #20). The different bacterial strains were incubated with β-LEAF alone and β-LEAF and cefazolin/cefoxitin/cefepime respectively. Fluorescence was monitored over 60 min. The y-axis represents cleavage rate of β-LEAF (measured as fluorescence change rate – milliRFU/min) normalized by bacterial O.D. (optical density) at 600 nm. Results are presented as the average of three independent experiments (each experiment contained samples in triplicates) and error bars represent the standard error.

The next set of investigations focussed on the β-LEAF assay to test multiple antibiotics simultaneously, to help predict the antibiotic activity profile for a particular bacterial isolate. For these studies we tested a sub-set of the isolates, the ATCC control strains (#1 and #2) and four isolates (#6, #18, #19, and #20) that produce appreciable amounts of β-lactamase as per both the β-LEAF assay and the nitrocefin test (Table 2). In addition to the first generation cephalosporin cefazolin, we used cefoxitin and cefepime, second and fourth generation cephalosporins respectively. Notably, cefepime is known to be more resistant to hydrolysis by β-lactamases [56,57]. In the β-LEAF and cefazolin or cefoxitin reactions, fluorescence was significantly reduced compared to β-LEAF alone reactions with all tested isolates (Figure 3). In contrast, for cefepime + β-LEAF reactions, the reduction in fluorescence was not as drastic as observed for the other two antibiotics, being 50% or even less (Figure 3). This incomplete reduction indicated that cefepime failed to compete efficiently with β-LEAF for the lactamase, despite its saturating concentration. Following this, cefepime is least likely to be inactivated by the β-lactamase, and thus predicted as likely to be most active for treatment among the three antibiotics tested. Bacteria-free (PBS only) control reactions are presented in Additional file 1: Figure S1.

To simplify interpretation, we calculated a ratio of the cleavage rate of β-LEAF in the presence of an antibiotic to cleavage rate of β-LEAF alone, for each antibiotic, for the different bacteria (Table 4). This ratio approaching ‘1’ indicates better activity of the tested antibiotic against the bacterial isolate in context of β-lactamase based resistance. Such an analysis is conceptually similar to the breakpoints values put forth by the CLSI and other regulatory authorities [41,42], where bacteria are classified as susceptible, intermediate or resistant to a given antimicrobial agent. This ratio in our method is meaningful only for isolates that produce significant amounts of β-lactamase. For other isolates, the difference in values when assayed with and without antibiotics respectively are negligible, and the ratios may give exaggerated results, such as for strain #2 (Table 4) and other isolates (data not shown). For such bacteria, the antibiotics may be considered active with regards to β-lactamase based resistance.

**Table 4:** Ratios from β-LEAF assays to assess activity of tested antibiotics in context of β-lactamase resistance

	***S. aureus *** **isolate**					
**Antibiotic**	**#1**	#2*	**#6**	**#18**	**#19**	**#20**
Cefazolin	0.11	0.55	0.08	0.13	0.12	0.36
Cefoxitin	0.11	0.64	0.09	0.12	0.12	0.30
Cefepime	0.68	0.44	0.80	0.58	0.47	0.66

Ratios were calculated as [Cleavage rate (β-LEAF + antibiotic)/Cleavage rate (β-LEAF alone)] using data depicted in Figure 3, for each antibiotic for the different bacteria tested, and rounded to two decimal points. Closer the value to ‘1’, more active an antibiotic predicted to be for the respective bacterial strain/isolate taking β-lactamase resistance into consideration.

NOTE: *For *isolates that show low cleavage rates* with β-LEAF (e.g. #2), there is negligible difference in values when antibiotics are included in the reaction, and the ratios may give exaggerated results. *For such strains, the antibiotics may be considered active/usable.*

### Comparison of E-test and β-LEAF assay results

Next, the antibiotic activity data for cefoxitin and cefepime from the fluorescence based β-LEAF assay was compared to antibiotic susceptibility determined using E-tests. We utilized the E-test an alternate AST method to determine antibiotic susceptibility conventionally. For *S. aureus*, cefoxitin is used as an oxacillin surrogate, and oxacillin resistance and cefoxitin resistance are equated [41]. Applying these criteria, #1, #2 and #6 were predicted as cefoxitin susceptible, while #18, #19 and #20 were predicted to have different degrees of resistance to cefoxitin (Table 5). However, #1, #6, #18, #19 and #20 were shown to be β-lactamase producers (Table 2, columns 2, 3 and 4), with the β-LEAF assay indicating cefoxitin to be less active (Figure 3, Table 4). All isolates were predicted to be susceptible to cefepime (Table 5), consistent with β-LEAF assay predictions, and with cefepime being stable to β-lactamases.

**Table 5:** Cefoxitin and Cefepime MIC (by E-test) for selected bacterial isolates

***S. aureus *** **isolate**	**Cefoxitin MIC (μg/ml)**	Cefoxitin AS*	**Cefepime MIC (μg/ml)**	Cefepime AS**
#1	3.0 ± 0.0	S	3.3 ± 0.3	S
#2	2.2 ± 0.4	S	1.7 ± 0.3	S
#6	3.0 ± 1.0	S	2.8 ± 0.7	S
#18	4.0 ± 1.0	I	2.0 ± 0.5	S
#19	6.0 ± 1.0	I	3.0 ± 0.6	S
#20	20.0 ± 2.3	R	7.0 ± 0.6	S

*The Cefoxitin Antibiotic Susceptibility (AS) was determined using the CLSI Interpretive Criteria for cefoxitin as an oxacillin surrogate [41].

≤ 4 μg/ml - Susceptible (S), ≥ 8 μg/ml- Resistant (R), values in between Intermediate (I).

**The Cefepime Antibiotic Susceptibility (AS) was determined using the CLSI Interpretive Criteria for cefepime [41].

≤ 8 μg/ml - Susceptible (S), 16 μg/ml - Intermediate (I), ≥ 32 μg/ml - Resistant (R)

The results are presented as an average of three independent experiments as Average ± Std. Error. The CLSI recommended quality control for MIC for *S. aureus,* ATCC 29213 (#1) was included each time, and showed MIC within the expected range for cefoxitin (1–4 μg/ml) and cefepime (1–4 μg/ml) respectively.

Cefoxitin and cefepime MICs with induced growth inoculum for these isolates were also determined (Additional file 3: Tables S2 and S3). Though MICs were marginally altered for some isolates with induced inoculum compared to standard inoculum, the antibiotic susceptibility interpretation was unaffected (Additional file 3: Tables S2 and S3).

### β-lactamase induction may not be necessary to perform β-LEAF assays

We also compared the effectiveness of the β-LEAF assay with induced growth cultures to un-induced cultures (Additional file 4: Figure S3). Growth in the presence of penicillin overnight serves to induce and enhance β-lactamase production, but adds another step. Without the induction step, the total turnover time from isolate obtained to antibiotic activity prediction would be only 1 hour. β-lactamase was readily detected even without induction, though at lower levels compared to induced cultures for some isolates (Additional file 4: Figure S3). Antibiotic susceptibility profiles were also similar for un-induced and induced bacteria (Additional file 4: Figure S3). As induction of lactamases may not be a pre-requisite for performing the β-LEAF assay, this result shows promise for extending the assay to rapid direct bio-specimen testing.

## Discussion

In order to combat bacterial infections effectively, the rapid identification of appropriate treatment modalities is critical [10]. Determination of antibiotic susceptibility and resistance are key to this process [8,9]. This report describes a rapid method to address these two aspects by exploiting the property of fluorescence quenching-dequenching. Although the sample numbers used in this study are too small for this method to be viewed as a robust dual assay at this stage, the results are promising. There are several mechanisms of bacterial resistance, both inherent and acquired, and production of β-lactamases, which enzymatically cleave and thereby inactivate β-lactam antibiotics, is a major pathway for antibiotic resistance and pathogen protection. The β-LEAF assay presented here focuses on this resistance mechanism. The strategy employs a molecular probe that is quenched until cleaved by the β-lactamase enzyme, following which fluorophores are dequenched and become fluorescent (Figure 1). The β-LEAF probe is designed to mimic β-lactam antibiotics and is thus sensitive to β-lactamases [49,50]. Owing to similarity in core structures, a β-lactam antibiotic and β-LEAF compete for the enzyme when present together [50]. The fluorescence readout therefore may report both presence of β-lactamases and β-lactam antibiotic activity. The competition between the probe and the β-lactam antibiotic results in reduced probe cleavage and hence diminished fluorescence, compared to when the probe is assayed alone (Figure 1). Using several-fold higher concentrations of the test β-lactam antibiotic, compared to the probe, enhances the likelihood that the antibiotic will be the preferred substrate of the lactamase in the competition reaction in the assay. The reduced fluorescence indirectly reflects the ability of the β-lactamase to bind and cleave the tested antibiotic (large difference = antibiotic can be readily bound and hence cleaved and inactivated). Notably, unlike growth based conventional AST methods, the end-point of the β-LEAF assay is not bacterial viability or differences in growth pattern. The read-out of the assay is fluorescence, which reflects probe cleavage due to the enzymatic activity of the β-lactamase. Importantly, the β-LEAF assay is rapid compared to the conventional growth based AST methods (1 h versus 20–24 h for disk diffusion/MIC conventionally or ~8 h with automated instruments).

The observation in Figure 2 of low to negligible fluorescence in β-LEAF + cefazolin reactions with all β-lactamase ‘positives’ (#1, #6, #18, #19, #20) suggests that cefazolin can be readily targeted and inactivated by the respective lactamases, and would be anticipated to be a less effective treatment option for these bacteria. An expectation of this assay is that the reduction in probe fluorescence in the presence of an antibiotic will be inversely proportional to its predicted activity against the pathogen. If fluorescence is completely reduced in the presence of an antibiotic, then the respective antibiotic can be readily cleaved and inactivated by β-lactamase. However, if despite the ‘saturating’ amount of antibiotic, some fluorescence increase reflecting probe cleavage is still observed (e.g. cefepime reactions in Figure 3), the lactamase may not be capable of effectively destroying the antibiotic, and the antibiotic predicted as likely to be active. In experiments with multiple antibiotics (Figure 3) a ratio of the cleavage rate of β-LEAF in presence of an antibiotic to the cleavage rate of β-LEAF alone, for each antibiotic tested, is shown in Table 4. For β-lactamase based resistance, the ratio of cleavage rates closer to 1 (Table 4) would indicate greater β-lactam antibiotic efficacy. With more rigorous testing from multiple data sets on a large number of isolates, cut-offs could be set up to develop the ratios as a ‘β-lactamase-based antibiotic activity/susceptibility index’ within specific limits. We recognize that there are a wide variety of lactamases, and note that with appropriate kinetic analysis (such as building on our previous study [50]), the approach presented here has the potential of characterizing the different lactamases.

The motivation for the choice of antibiotics used in this initial study was to test three different generations of cephalosporin antibiotics. Cephalosporins are a standard treatment for skin and soft-tissue infections [58,59]. Cefazolin is the most commonly used first generation cephalosporin. Cefoxitin is a cephamycin antibiotic, classified as a second-generation cephalosporin. The importance of testing with cefoxitin is also increased because it is routinely used as an oxacillin-surrogate routinely for susceptibility testing [41] and MRSA phenotype prediction [60-64]. Cefepime is a fourth generation cephalosporin that is designed to have better stability against β-lactamases [56,57]. Consistent with this, the β-LEAF assay accurately identified cefepime as the most resistant to the β-lactamase(s) in our experiments (Figure 3, Table 4).

Interestingly, the cefazolin disk diffusion results indicated all isolates as cefazolin susceptible, while analyses from the β-LEAF assays predicted that cefazolin would be less active for five of the isolates (#1, #6, #18, #19, #20) (Table 2 - columns 5 and 6). At the same time, the zone edge test applied to disk diffusion plates [55] matched the β-lactamase prediction from both the nitrocefin tests and β-LEAF assay for these isolates (Table 2- columns 2, 3 and 4). Similarly, while the E-tests suggested isolates #1 and #6 to be cefoxitin susceptible (and #18, #19, #20 to have different degrees of resistance to cefoxitin) (Table 5), the β-LEAF assay predicted that cefoxitin could be inactivated by these isolates, by virtue of lactamase production (Figure 3). Notably, discrepancies between susceptibility prediction and antibiotic efficacy can occur. Conventional AST methods such as disk diffusion and MIC determination may occasionally fail to take resistance into account and/or misreport antibiotic susceptibility, and special tests may be required to detect resistance mechanisms [44-47]. Another example is that the CLSI recommends performing tests to detect β-lactamase production on staphylococci for which penicillin zone diameters are ≥ 29 mm or MIC ≤ 0.12 μg/ml, before reporting isolates as susceptible [41,42], which suggests that taking β-lactamase production into consideration additionally may be important. Thus, taken as a whole, the results of the standard tests and β-LEAF are consistent when considering lactamase production along with disk diffusion or MIC results. By providing a rapid mode to test lactamase production as well as help predict antibiotic activity, the β-LEAF assay could prove to be advantageous and potentially minimize the need for additional testing.

The overall agreement between standard CLSI recommended methodologies and the proposed assay in this work for β-lactamase detection and antibiotic activity/susceptibility is encouraging, particularly in view of the fact that β-LEAF assay provides these results from a rapid (1 h) assay. When validated with a large sample number, the assay could be adapted as a rapid diagnostic of antibiotic susceptibility, and serve as a useful adjunct in management of antibiotic resistance [10].

An important aspect is that in cases of high bacterial burdens and/or heteroresistance the ‘inoculum effect’ can affect antibiotic activity [35,65-67]. Though cephalosporins are used as standard treatment, they can be hydrolyzed by β-lactamases at high inocula (‘inoculum effect’), resulting in clinical failures [33-40]. Conventional ASTs typically utilize 5*10^5^ CFU/ml as standard test inoculums [41,42]. Koing et al. studied the efficacy of several antibiotics against *Escherichia coli* and *S. aureus,* and cited much higher bacterial numbers in infections compared to numbers used in standard susceptibility tests as a major reason for predicted antibiotic susceptibility not matching with observed efficacy [68]. Pus and infected peritoneal samples, for example, contain an average of 2*10^8^ CFU/ml, a concentration 400 times higher than the inocula used for standard conventional ASTs [68]. The β-LEAF assay is compatible with usage of high bacterial numbers (i.e. ~10^8^ CFU and higher), by virtue of which it may facilitate assessments at clinically relevant numbers based on infection sites. Some conventional AST methods, such as those relying on turbidometric detection of bacterial growth, may not be able to utilize higher bacterial numbers as the starting inoculum.

Although PCR-based diagnostics have been employed to detect antibiotic resistance factors relatively rapidly [69-72], the presence of a gene does not necessarily reflect expression of the protein (e.g. enzyme), actually responsible for conferring resistance. For instance, *Bacillus anthracis* contains genes for lactamases *bla1* and *bla2*, but usually resistance is not observed [73]. In the current study also, despite the different diagnostic methodologies for β-lactamase enzyme production being consistent (nitrocefin disk test, zone edge test and the β-LEAF assay), the *blaZ* genotype did not match for some of the isolates (Table 2). In these isolates (e.g. #9, #15) no β-lactamase production was observed, although they contained the gene for β-lactamase (*blaZ*). Thus, investigating the protein resistance factor phenotypically can be of value. Rapid determination of functional β-lactamase and its correlation to antibiotic activity/usability by assaying for enzyme activity is a distinctive feature of the β-LEAF assay.

## Conclusions

This study reports a fluorescence quenching-dequenching guided method for rapid β-lactamase detection and prediction of antibiotic activity in the context of β-lactamase. The initial results with standard ATCC bacterial strains and clinical isolates are encouraging, though further validation in a large number of isolates is required. The technology merits further rigorous and broader investigations with bacterial strains, antibiotics and direct biological samples to be a viable routine methodology. This requires the development of more sensitive probes and perhaps some novel engineering, which are currently being evaluated. The β-LEAF assay results are available within one hour and in the long-term such timely assessment could be used to guide treatment options for a particular infection, to ensure adequate therapy while avoiding unnecessary over- and under-prescription of antibiotics.

Because of the focus on β-lactamase, the current study has concentrated on β-lactam based probe constructs. However, the approach represents an optical platform using photoactivatable constructs that can be adapted for several targets that might confer antibiotic resistance. An interesting area of exploration is the use of the same technology for therapy where the constructs could be modified to specifically target β-lactamase resistant bacteria [49], in a variation of photodynamic therapy [74,75] that has shown promise in several indications of infections.

## Competing interests

The authors declare that they have no competing interests.

## Authors’ contributions

SK contributed to the design, conduct and analyses of experiments, and the writing and preparation of the manuscript. UWS contributed to the early conception, design and conduct of the β-LEAF assay. XZ synthesized the molecular probe and contributed to the early experiments and data analyses. GJN contributed to the study design, data interpretation and manuscript writing. TH contributed to the study conception and design, writing of the manuscript and overall supervision. All authors read and approved the final manuscript.

## Supplementary Material

Additional file 1: Figure S1 β-LEAF cleavage rates for ATCC control strains and bacteria free controls. Data from the two ATCC *S. aureus* control strains [known β-lactamase producer ATCC 29213 (#1) and non-producer ATCC 25923 (#2)] and PBS only control, with three antibiotics (cefazolin, cefoxitin and cefepime) is presented. The different samples were incubated with β-LEAF (probe) alone or β-LEAF and respective antibiotic, and fluorescence was monitored over 60 min. The y-axis represents the cleavage rate of β-LEAF (measured as fluorescence change rate – milliRFU/min) (Bacterial O.D. is not accounted for here). Results are presented as the average of four independent experiments (each experiment contained samples in triplicates) and error bars represent the standard error. Click here for file

Additional file 2: Figure S2 Standard Disk diffusion assay to determine cefazolin susceptibility and zone edge test for β-lactamase detection. Representative Disk diffusion plates for the control strains *S. aureus* ATCC 29213 (#1) and ATCC 25923 (#2) are shown, with the cefazolin disk at the centre of the plate. The clear zone of inhibition and zone edges are indicated. #1 was used as a positive control for the zone edge test (sharp edge) and #2 as a negative control (fuzzy edge), following CLSI guidelines. Click here for file

Additional file 3: Table S1 Comparison of cefazolin disk diffusion results for ‘standard growth’ and ‘induced growth’ bacterial cultures. **Table S2.** Comparison of cefoxitin MIC results (by E-test) for ‘standard growth’ and ‘induced growth’ bacterial cultures. **Table S3.** Comparison of cefepime MIC results (by E-tests) for ‘standard growth’ and ‘induced growth’ bacterial cultures. Click here for file

Additional file 4: Figure S3 β-lactamase induction is not necessary prior to performing β-LEAF assays for *S. aureus*. β-LEAF assays were performed with the two ATCC *S. aureus* control strains (positive control #1 and negative control #2) and four *S. aureus* clinical isolates that showed substantial β-lactamase production (#6, #18, #19, #20), using both induced and un-induced growth cultures. (i) denotes ‘induced’ growth bacteria, grown in the presence of a penicillin disk overnight to induce and enhance β-lactamase production; (ui) denotes ‘un-induced’ bacteria, grown on plain plates without any inducing antibiotic. The different bacteria were incubated with β-LEAF alone and β-LEAF and cefazolin/cefoxitin/cefepime respectively. Fluorescence was monitored over 60 min. The y-axis represents cleavage rate of β-LEAF (measured as fluorescence change rate – milliRFU/min) normalized by bacterial O.D. (optical density) at 600 nm. Results are presented as the average of three independent experiments (each experiment contained samples in triplicates) and error bars represent the standard error. Click here for file
