# Supplementary figures and images for: Rapid optical determination of β-lactamase and antibiotic activity
Source: BMC Microbiol. 2014 Apr 4;14:84. doi: 10.1186/1471-2180-14-84 (PMC4234275; doi:10.1186/1471-2180-14-84)

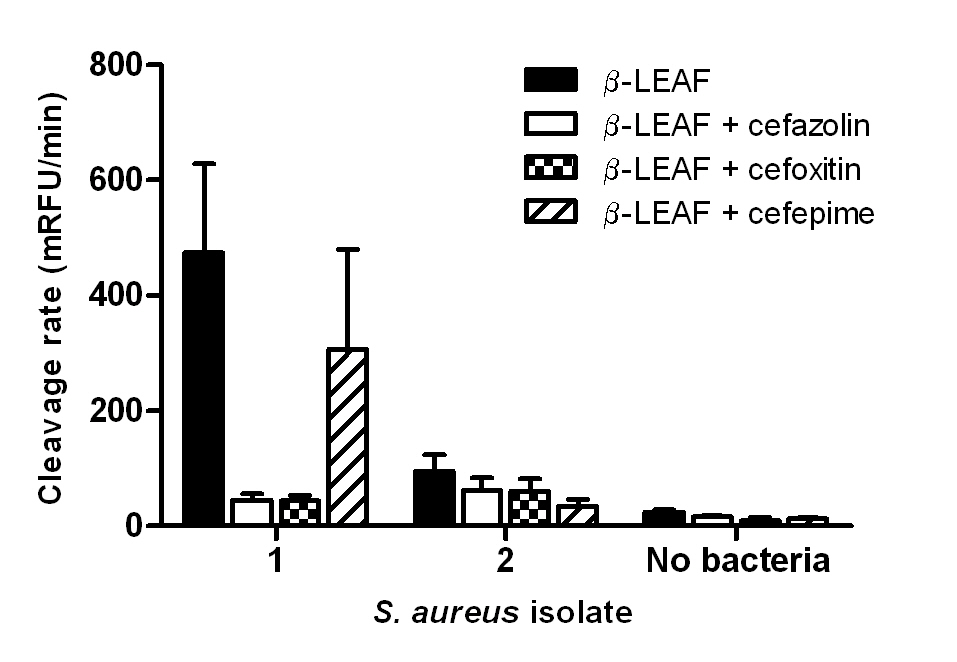

Supplement: Additional file 1: Figure S1 — β-LEAF cleavage rates for ATCC control strains and bacteria free controls. Data from the two ATCC S. aureus control strains [known β-lactamase producer ATCC 29213 (#1) and non-producer ATCC 25923 (#2)] and PBS only control, with three antibiotics (cefazolin, cefoxitin and cefepime) is presented. The different samples were incubated with β-LEAF (probe) alone or β-LEAF and respective antibiotic, and fluorescence was monitored over 60 min. The y-axis represents the cleavage rate of β-LEAF (measured as fluorescence change rate – milliRFU/min) (Bacterial O.D. is not accounted for here). Results are presented as the average of four independent experiments (each experiment contained samples in triplicates) and error bars represent the standard error. [file 1471-2180-14-84-S1.jpeg]

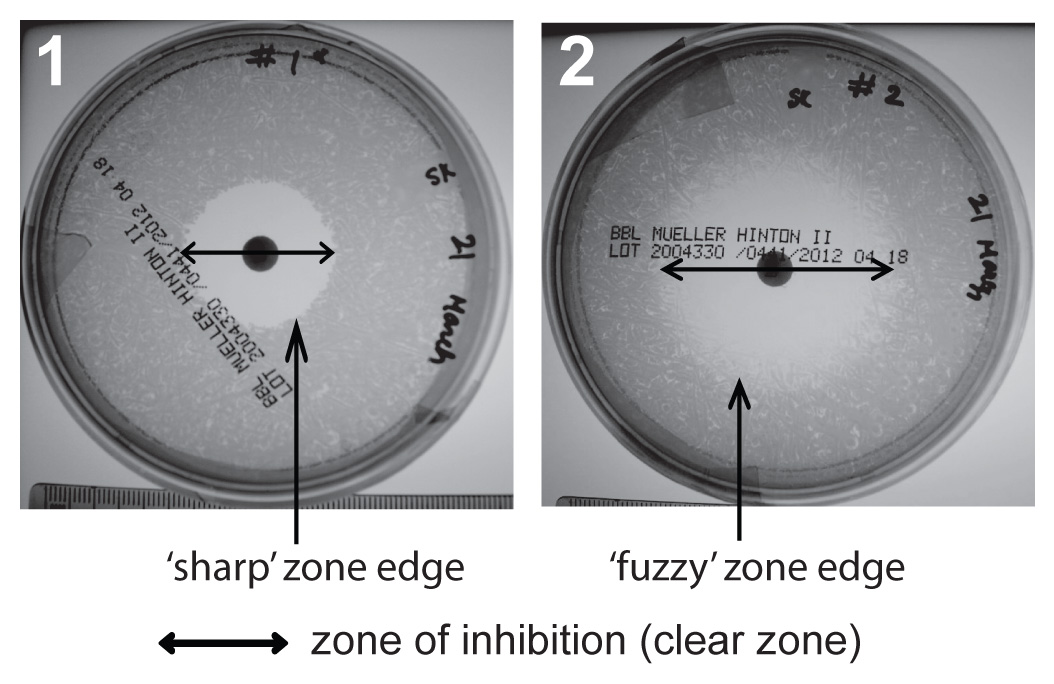

Supplement: Additional file 2: Figure S2 — Standard Disk diffusion assay to determine cefazolin susceptibility and zone edge test for β-lactamase detection. Representative Disk diffusion plates for the control strains S. aureus ATCC 29213 (#1) and ATCC 25923 (#2) are shown, with the cefazolin disk at the centre of the plate. The clear zone of inhibition and zone edges are indicated. #1 was used as a positive control for the zone edge test (sharp edge) and #2 as a negative control (fuzzy edge), following CLSI guidelines. [file 1471-2180-14-84-S2.jpeg]

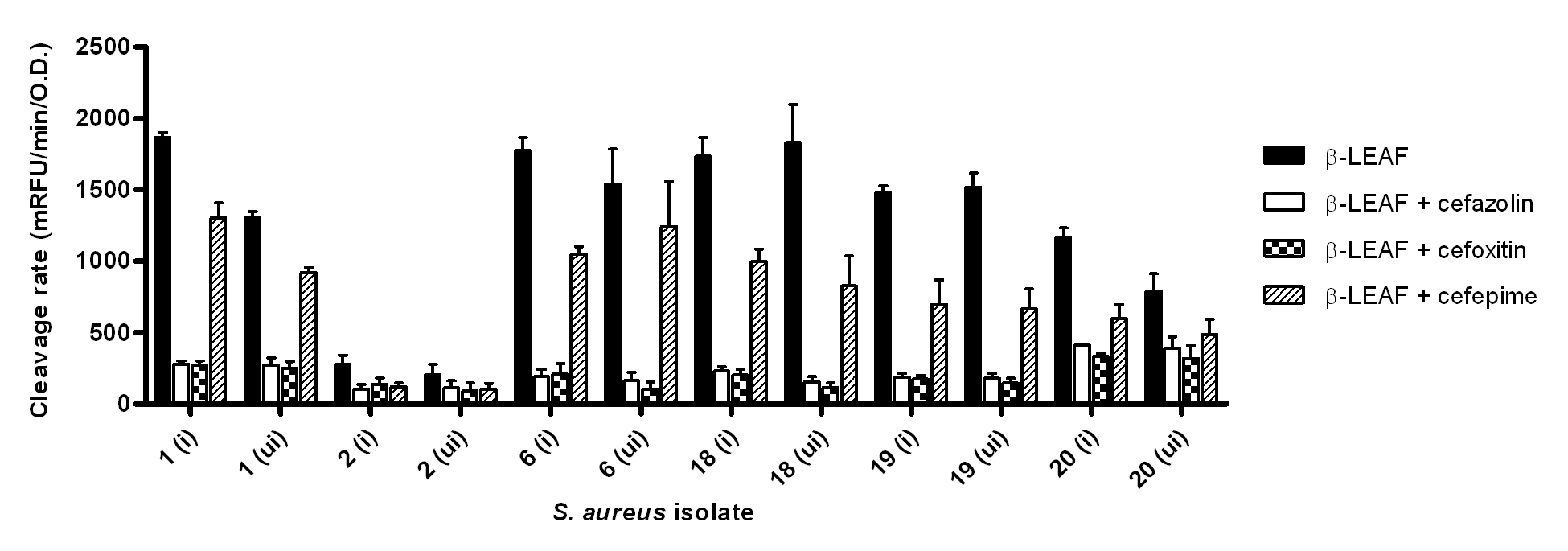

Supplement: Additional file 4: Figure S3 — β-lactamase induction is not necessary prior to performing β-LEAF assays for S. aureus. β-LEAF assays were performed with the two ATCC S. aureus control strains (positive control #1 and negative control #2) and four S. aureus clinical isolates that showed substantial β-lactamase production (#6, #18, #19, #20), using both induced and un-induced growth cultures. (i) denotes ‘induced’ growth bacteria, grown in the presence of a penicillin disk overnight to induce and enhance β-lactamase production; (ui) denotes ‘un-induced’ bacteria, grown on plain plates without any inducing antibiotic. The different bacteria were incubated with β-LEAF alone and β-LEAF and cefazolin/cefoxitin/cefepime respectively. Fluorescence was monitored over 60 min. The y-axis represents cleavage rate of β-LEAF (measured as fluorescence change rate – milliRFU/min) normalized by bacterial O.D. (optical density) at 600 nm. Results are presented as the average of three independent experiments (each experiment contained samples in triplicates) and error bars represent the standard error. [file 1471-2180-14-84-S4.jpeg]
