# Supplementary material for: Rapid optical determination of β-lactamase and antibiotic activity
Source: BMC Microbiol. 2014 Apr 4;14:84. doi: 10.1186/1471-2180-14-84 (PMC4234275; doi:10.1186/1471-2180-14-84)
Supplement: Additional file 3: Table S1 — Comparison of cefazolin disk diffusion results for ‘standard growth’ and ‘induced growth’ bacterial cultures. Table S2. Comparison of cefoxitin MIC results (by E-test) for ‘standard growth’ and ‘induced growth’ bacterial cultures. Table S3. Comparison of cefepime MIC results (by E-tests) for ‘standard growth’ and ‘induced growth’ bacterial cultures. [file 1471-2180-14-84-S3.doc]

**Additional file 3**

**Table S1: Comparison of cefazolin disk diffusion results for ‘standard growth’ and ‘induced growth’ bacterial cultures.**

| ***S. aureus* isolate** | **STANDARD INOCULUM** | | | | **INDUCED INOCULUM** | | | |
| --- | --- | --- | --- | --- | --- | --- | --- | --- |
|  | **Zone of inhibition diameter (mm)** | **AS ^*^** | **Zone edge^&^** | **Interpretation as per Zone edge test criteria^&^** | **Zone of inhibition diameter (mm)** | **AS ^*^** | **Zone edge** | **Interpretation as per Zone edge test criteria^&^** |
| **#1** | 21.5 ± 1.0 | S | sharp | β | 23.5 ± 3.5 | S | sharp | β |
| **#2** | 31.0 ± 1.0 | S | fuzzy | - | 29.5 ± 0.5 | S | fuzzy | - |
| **# 6** | 36.5 ± 0.5 | S | sharp | β | 41.0 ± 0.0 | S | sharp | β |
| **# 18** | 33.5 ± 0.5 | S | sharp | β | 33.0 ± 0.0 | S | sharp | β |
| **# 19** | 31.0 ± 0.0 | S | sharp | β | 31.5 ± 0.5 | S | sharp | β |
| **# 20** | 20.5 ± 0.5 | S | sharp | β | 21.0 ± 2.0 | S | sharp | β |

Results using both standard inoculum (grown in absence of any antibiotics) and induced cultures (grown in presence of penicillin disks) are shown for selected isolates.

**^*^**The Antibiotic Susceptibility (AS) was determined using the CLSI Zone Diameter Interpretive Criteria for Cefazolin Disk Diffusion [1]:

≤ 14 mm: Resistant (R); 15-17 mm: Intermediate (I); ≥ 18 mm: Susceptible (S)

The results shown are averages of at least two independent experiments, and are presented as Average ± Standard Error. The CLSI recommended quality control strain ATCC 25923 (#2) was included each time and gave zone of inhibition diameter within the expected range (29-35mm).

**^&^** The **zone edge test** was also applied, and the edge of the zone of inhibition was observed. *S. aureus* ATCC 29213 (#1) was used as a positive for the zone edge test (sharp edge), and ATCC 25923 (#2) as negative control (fuzzy edge). ‘**β’ denotes β -lactamase producing strain**

**Table S2: Comparison of cefoxitin MIC results (by E-test) for ‘standard growth’ and ‘induced growth’ bacterial cultures**

| ***S. aureus* isolate** | **STANDARD INOCULUM** | | **INDUCED INOCULUM** | |
| --- | --- | --- | --- | --- |
|  | **Cefoxitin MIC (μg/ml)** | **AS *** | **Cefoxitin MIC (μg/ml)** | **AS *** |
| **#1** | 3.0 ± 0.0 | S | 3.0 ± 0.0 | S |
| **#2** | 2.2 ± 0.4 | S | 2.2 ± 0.2 | S |
| **# 6** | 3.0 ± 1.0 | S | 3.0 ± 0.0 | S |
| **# 18** | 4.0 ± 1.0 | I | 4.0 ± 0.0 | I |
| **# 19** | 6.0 ± 1.0 | I | 6.0 ± 2.0 | I |
| **# 20** | 20.0 ± 2.3 | R | 20 ± 4.0 | R |

Results using both standard inoculum (grown in absence of any antibiotics) and induced cultures (grown in presence of penicillin disks) are shown for selected isolates.

*The Antibiotic Susceptibility (AS) was determined using the CLSI Interpretive Criteria for cefoxitin as an oxacillin surrogate [1]:

≤ 4 μg/ml Susceptible (S), ≥ 8 μg/ml Resistant (R),values in between Intermediate (I).

The results are presented as an average of at least two independent experiments as Average ± Std. Error.

The CLSI recommended quality control for MIC for *S. aureus,* ATCC 29213 (#1) was included each time, and showed MIC within the expected range (1-4 μg/ml).

**Table S3: Comparison of cefepime MIC results (by E-tests) for ‘standard growth’ and ‘induced growth’ bacterial cultures**

| ***S. aureus* isolate** | **STANDARD INOCULUM** | | **INDUCED INOCULUM** | |
| --- | --- | --- | --- | --- |
|  | **Cefepime MIC (μg/ml)** | **AS **** | **Cefepime MIC (μg/ml)** | **AS **** |
| #1 | 3.3 ± 0.3 | S | 3.5 ± 0.5 | S |
| #2 | 1.7 ± 0.3 | S | 2.5 ± 0.5 | S |
| # 6 | 2.8 ± 0.7 | S | 3.5 ± 0.5 | S |
| # 18 | 2.0 ± 0.5 | S | 3.0 ± 1.0 | S |
| # 19 | 3.0 ± 0.6 | S | 3.5 ± 0.5 | S |
| # 20 | 7.0 ± 0.6 | S | 7.5 ± 0.5 | S |

**The Antibiotic Susceptibility (AS) was determined using the CLSI Interpretive Criteria for cefepime [1]:

≤ 8 μg/ml Susceptible (S), 16 μg/ml Intermediate (I), ≥ 32 μg/ml Resistant (R)

The results are presented as an average of at least two independent experiments as Average ± Std. Error.

The CLSI recommended quality control for MIC for *S. aureus,* ATCC 29213 (#1) was included each time, and showed MIC within the expected range (1-4 μg/ml).

**REFERENCES**

1. CLSI, *Performance standards for antimicrobial susceptibility testing: Twenty-second informational supplement; M100-S22.* Clinical and Laboratory Standards Institute, Wayne, Pennsylvania, USA, 2012.
